# Biochemical Evaluation of Bone Health in Children with Phenylketonuria, Organic Acidemias, and Glycogen Storage Diseases Under Dietary Management

**DOI:** 10.3390/nu18172751

**Published:** 2026-08-22

**Authors:** Sabire Gokalp, Hatice Pasaoglu

**Affiliations:** 1 Department of Pediatric Metabolic Disorders, Etlik City Hospital, Ankara 06170, Turkey; 2 Department of Medical Biochemistry, Faculty of Medicine, Gazi University, Ankara 06560, Turkey; pasaogluh@yahoo.com

**Keywords:** inherited metabolic disorders, bone health, bone turnover markers, phenylketonuria, organic acidemias, glycogen storage diseases, dietary management

## Abstract

**Background**: Inherited metabolic disorders (IMDs) are a heterogeneous group of genetic diseases, some of which require lifelong disease-specific dietary management. Children with phenylketonuria (PKU), organic acidemias (OAs), and glycogen storage diseases (GSDs) are particularly susceptible to nutritional imbalances and metabolic disturbances that may adversely affect bone metabolism and skeletal health. However, data regarding bone turnover markers and bone health in these pediatric IMD populations remain limited. **Objective**: This study aimed to evaluate bone metabolism by assessing biochemical parameters, bone turnover markers, and bone mineral density in children with PKU, OA, and GSD receiving long-term dietary treatment. **Methods**: This cross-sectional study included 95 children, comprising 25 patients with PKU, 20 patients with organic acidemias, 20 patients with glycogen storage diseases, and 30 healthy age-matched controls. Demographic characteristics, anthropometric measurements, and dietary intake were recorded. Biochemical evaluation included serum calcium, phosphorus, alkaline phosphatase (ALP), parathyroid hormone (PTH), and 25-hydroxyvitamin D levels. Bone turnover was assessed using C-terminal telopeptide of type I collagen (CTX), procollagen type I N-terminal propeptide (P1NP), and osteocalcin (OC) levels. Bone mineral density was evaluated by dual-energy X-ray absorptiometry (DXA). **Results**: Age and sex distributions were similar among the groups. Height was lower in OA than in controls and PKU, and in GSD than in PKU; weight and BMI were lower in both OA and GSD than in controls and PKU (*p* < 0.05). Significant differences were observed in dietary energy and macronutrient intake according to disease-specific nutritional regimens. Serum calcium, phosphorus, and 25-hydroxyvitamin D levels were significantly lower in all IMD groups compared with healthy controls (*p* < 0.001). ALP and PTH levels were significantly higher in PKU, OA, and GSD patients than in controls (*p* < 0.001). Among bone turnover markers, P1NP and osteocalcin levels significantly differed among groups, with the highest values observed in PKU patients (*p* = 0.006 and *p* = 0.028, respectively). CTX levels did not significantly differ among the groups (*p* = 0.183). DXA Z-scores were significantly lower in all IMD groups than in controls, with the lowest mean Z-scores observed in GSD patients. **Conclusions**: Children with PKU, OA, and GSD receiving long-term dietary treatment exhibited significant alterations in bone metabolism, characterized by lower vitamin D, calcium, and phosphorus levels, higher ALP and PTH concentrations, differences in bone formation markers, and lower DXA Z-scores compared with controls. Significant differences were observed in the bone formation markers P1NP and osteocalcin, whereas CTX levels remained comparable among the study groups. These findings underscore the importance of regular assessment of bone health and optimization of nutritional management in children under dietary treatment, while acknowledging that disease-specific mechanisms of skeletal involvement may differ among these conditions.

## 1. Introduction

Inherited metabolic disorders (IMDs) comprise a heterogeneous group of genetic disorders characterized by profound biochemical disruptions that manifest early in life and necessitate intricate, often lifelong medical and dietary management [1,2,3]. Historically, the primary objective of clinical management was centered on the prevention of acute metabolic crises and the mitigation of irreversible neurological damage through newborn screening and immediate therapeutic intervention [2,4,5]. While specialized dietary regimens and pharmacological advancements have revolutionized patient outcomes and significantly extended life expectancy into adulthood, they have simultaneously unveiled a new spectrum of long-term chronic complications that were previously unrecognized [1,5]. Among these emerging concerns, impaired bone health has become an important long-term complication in several inherited metabolic disorders, particularly PKU, OA, and GSD [6]. The etiology of bone disease in this population is increasingly recognized as multifactorial, representing a complex interplay between the underlying genetic defect, the restrictive nature of therapeutic diets, and secondary metabolic imbalances such as chronic acidosis, systemic nutritional deficiencies, or altered endocrine signaling [6,7].

Phenylketonuria (PKU; OMIM 261600), resulting from a deficiency in the enzyme phenylalanine hydroxylase (PAH), is the most prevalent IMD requiring a lifelong phenylalanine (Phe)-restricted diet [8,9]. While this regimen successfully prevents severe intellectual disability, concerns regarding its impact on the skeleton have persisted for decades, with many patients exhibiting significantly lower mean BMD Z-scores compared to healthy reference populations across all age groups [4,6]. One critical hypothesized mechanism is the high potential renal acid load (PRAL) of synthetic amino acid (AA) protein substitutes, which are essential for providing non-Phe protein equivalents but can induce chronic, undiagnosed metabolic acidosis [10,11]. This state of acidosis is thought to increase the urinary excretion of calcium and magnesium, causing the skeleton to act as a buffer reservoir and leading to subsequent demineralization [3,11]. Furthermore, recent cellular studies have highlighted the role of spontaneous osteoclastogenesis in PKU, where elevated Phe levels and activated T cells promote osteoclast differentiation through the RANKL/OPG pathway, shifting the homeostatic balance toward increased bone resorption [9,12]. Additionally, the reliance on synthetic mixtures can lead to suboptimal intake or bioavailability of essential bone-forming micronutrients like Vitamin D, phosphorus, and zinc, further compromising peak bone mass attainment [13,14,15].

Organic acidemias (OAs), including propionic acidemia (PA; OMIM 606054) and methylmalonic acidemia (MMA; OMIM 251000), present even more severe risks to skeletal integrity. Management of propionic acidemia (PA) and methylmalonic acidemia (MMA) requires lifelong dietary restriction of the propiogenic amino acids isoleucine, valine, methionine, and threonine through carefully controlled natural protein intake, while adequate protein requirements are maintained with precursor-free medical formulas. High-energy nutritional support is also required to prevent catabolism. Despite these interventions, long-term dietary treatment may predispose patients to inadequate intake or altered bioavailability of calcium, vitamin D, and other micronutrients essential for normal bone matrix formation and mineralization. Clinical reports have documented a strikingly high prevalence of osteopenia and osteoporosis in this population, with children and young adults frequently presenting with pathological fractures such as vertebral crush fractures or supracondylar femoral fractures occurring with minimal or no trauma [16,17]. In classical MMA, secondary systemic complications, most notably chronic renal failure (CRF) and delayed pubertal maturation, further exacerbate the risk of skeletal fragility by interfering with the activation of vitamin D and overall mineral homeostasis [18].

Glycogen storage diseases (GSDs), particularly hepatic types such as Type Ia (OMIM 232200), Type III (OMIM 232400), and Type IX, involve enzyme deficiencies that disrupt normal glucose homeostasis and glycogen metabolism [7]. Management of hepatic glycogen storage diseases relies on maintaining normoglycemia through frequent meals, avoidance of prolonged fasting, and, in many patients, uncooked cornstarch therapy. Patients with GSD type I additionally require restriction of fructose and galactose, whereas those with GSD types III, VI, and IX, are generally managed with higher protein intake to support gluconeogenesis and maintain metabolic stability. Although these dietary interventions improve metabolic control, long-term nutritional management together with the underlying metabolic disorder may adversely affect bone metabolism and skeletal health. In GSD Type I, chronic lactic acidosis, hyperlipidemia, and metabolic instability are key factors that interfere with bone mineralization and mineral accretion. Although prepubertal patients may initially show normal BMD, significant reductions are frequently observed in adolescents and adults [19,20]. In GSD Type III, which often involves muscular compromise, the muscle–bone relationship is a determining factor for skeletal health; reduced mechanical stimulation from hypotrophic muscles and decreased muscle force lead to a failure in adapting bone strength to functional requirements [21]. This concept, known as the mechanostat, posits that bone development is not just about mass accumulation but functional stability related to the force of the muscles attached to it. Furthermore, reduced levels of anabolic agents such as IGF-1 and insulin in GSD III patients are strongly associated with impaired bone remodeling and decreased mature osteoblast activity [19,21,22].

While dual-energy X-ray absorptiometry (DXA) remains the clinical “gold standard” for assessing BMD, its interpretation in pediatric and adult IMD patients is often hampered by short stature, delayed bone maturation, and the fact that it primarily reflects static mineral status rather than the dynamic processes of bone turnover [7,10,20,23]. Consequently, there is an increasing clinical imperative for the biochemical evaluation of bone health to assess remodeling dynamics through non-invasive biomarkers. Biomarkers of bone formation, such as bone-specific alkaline phosphatase (BALP), osteocalcin (OC), and procollagen type 1 N-terminal propeptide (P1NP), provide critical insights into osteoblastic activity and are often found to be inappropriately low or uncoupled in IMD patients. Conversely, resorption markers like collagen type 1 C-terminal telopeptide (CTX) and deoxypyridinoline (DPD) reflect osteoclast-mediated bone breakdown. Integrating these biochemical indicators with standard densitometry is essential for the early detection of remodeling imbalances and for monitoring the long-term impact of restrictive therapeutic diets on the skeletal system in IMD patients [23].

Although these disorders differ substantially in their underlying pathophysiology, they share long-term dietary management as a cornerstone of treatment, making the evaluation of bone health clinically relevant across these conditions.

The primary objective of this study was to provide a comprehensive biochemical evaluation of bone metabolism in children with PKU, OA, and GSD receiving specialized dietary treatment through the assessment of conventional biochemical parameters and bone turnover markers.

## 2. Materials and Methods

### 2.1. Study Design and Participants

This cross-sectional study was conducted to evaluate bone health in children with IMDs receiving long-term disease-specific dietary treatment. The study aimed to investigate differences in bone metabolism associated with disease-specific dietary regimens using biochemical and radiological parameters. A total of 95 participants were included, comprising 25 patients with phenylketonuria (PKU), 20 patients with organic acidemias (OAs), 20 patients with glycogen storage disease (GSD), and 30 healthy controls. Patients aged between 3 and 18 years who had been receiving dietary treatment since diagnosis and had regular clinical follow-up were eligible for inclusion. None of the patients with phenylketonuria included in this study were receiving sapropterin therapy. Patients with chronic conditions or medication use potentially affecting bone metabolism, as well as those lacking regular follow-up data, were excluded from the study. Patients with chronic kidney disease, endocrine disorders, glucocorticoid use, or a history of fractures were excluded from the study. All participants had serum creatinine levels and estimated glomerular filtration rates within the age-appropriate reference ranges.

### 2.2. Clinical and Dietary Assessment

Demographic and clinical data, including age, sex, age at diagnosis, disease duration, and dietary treatment characteristics, were obtained from medical records. Dietary intake and dietary adherence were also evaluated. The food records were reviewed by a pediatric metabolic disease physician and a dietitian. A three-day food consumption record, including one weekend day, was obtained from each participant. Energy and nutrient intakes were analyzed using the Turkish version of the Nutrition Information System software (BeBiS; version 8.2; Ebispro for Windows, Stuttgart, Germany) and its integrated food-composition database. Disease-specific medical formulas and nutritional supplements were included in the analysis according to the amounts consumed and their nutrient composition, and their contributions were incorporated into the total daily nutrient intake. Dietary adherence was assessed by comparing the recorded intake with each patient’s individualized disease-specific dietary prescription, including prescribed nutrient intake, use of medical formulas, and meal pattern, as applicable. Because dietary prescriptions differed among the metabolic disorders, a single numerical adherence threshold was not applied across all patient groups.

### 2.3. Biochemical and Bone Turnover Marker Analysis

Biochemical parameters of bone metabolism were assessed using fasting serum samples obtained as part of routine clinical evaluation. Serum calcium, phosphorus, and alkaline phosphatase (ALP) were measured by standard automated clinical chemistry methods. Serum parathyroid hormone (PTH), 25-hydroxyvitamin D [25(OH)D], procollagen type I N-terminal propeptide (P1NP), C-terminal telopeptide of type I collagen (CTX), and osteocalcin concentrations were determined at the Central Biochemistry Laboratory of Gazi University Health Research and Application Center using Roche Elecsys electrochemiluminescence immunoassays (ECLIAs) on a cobas pro e801 analyzer (Roche Diagnostics, Mannheim, Germany), according to the manufacturer’s instructions. CTX was used as a marker of bone resorption, whereas P1NP and osteocalcin were used as markers of bone formation.

### 2.4. Bone Mineral Density Assessment

Bone mineral density (BMD) was assessed by dual-energy X-ray absorptiometry (DXA) at the lumbar spine (L1–L4). Results were expressed as age- and sex-adjusted Z-scores according to the manufacturer’s pediatric reference database.

### 2.5. Statistical Analysis

Descriptive statistics were presented as mean ± standard deviation, median, minimum–maximum values, frequencies, and percentages. Distribution of variables was assessed using the Kolmogorov–Smirnov and Shapiro–Wilk tests. Parametric variables were compared using one-way ANOVA followed by Tukey’s post hoc test. Nonparametric variables were analyzed using the Kruskal–Wallis test followed by pairwise Mann–Whitney U tests with Holm adjustment for multiple comparisons. Categorical variables were compared using the chi-square test. Statistical analyses were performed using SPSS version 27.0, and a *p*-value < 0.05 was considered statistically significant.

## 3. Results

A total of 95 participants were included in the study, including 25 children with phenylketonuria (PKU), 20 with organic acidemias (17 methylmalonic acidemia and three propionic acidemia), 20 with glycogen storage diseases (13 GSD type Ia, three GSD type III, two GSD type VI, and two GSD type IX), and 30 healthy controls. The overall median age of the cohort was 7 years (range: 3–16 years), with a mean age of 7.0 ± 2.7 years. Age and sex distributions were comparable among the groups, and no statistically significant differences were observed (*p* = 0.104 and *p* = 0.681, respectively) (Table 1).

The mean age was 7.22 ± 2.39 years in the control group, 7.92 ± 3.32 years in the PKU group, 6.10 ± 2.34 years in the OA group, and 6.53 ± 2.13 years in the GSD group. The female to male ratios were similar across all study groups. The mean age at diagnosis significantly differed among the metabolic disease groups (*p* < 0.001). Patients with GSD had the latest diagnosis (16.15 ± 11.33 months), whereas PKU patients had the earliest diagnosis (1.20 ± 0.50 months). OA patients were diagnosed at an intermediate age (4.10 ± 2.88 months).

Anthropometric evaluation revealed significant differences among the groups. Height was lower in OA than in controls and PKU, while GSD differed only from PKU (*p* = 0.002). Weight and BMI were lower in OA and GSD than in controls and PKU (*p* < 0.001 and *p* = 0.001, respectively). No significant differences were detected between the control and PKU groups regarding anthropometric measurements (Table 2).

Detailed dietary intake data for each study group are presented in Supplementary Table S1. Daily energy intake was significantly higher in OA (96.8 ± 16.0 kcal/kg/day) and GSD patients (93.9 ± 14.3 kcal/kg/day) than in controls (75.3 ± 7.6 kcal/kg/day) and PKU patients (72.8 ± 9.0 kcal/kg/day) (*p* < 0.001). Total protein intake also differed significantly among the groups (*p* < 0.001). The highest protein intake was observed in the GSD group (2.25 ± 0.27 g/kg/day), followed by OA patients (1.94 ± 0.38 g/kg/day), PKU patients (1.38 ± 0.19 g/kg/day), and controls (1.04 ± 0.05 g/kg/day). Carbohydrate intake also differed significantly among the groups (*p* < 0.001), with the highest intake observed in OA patients (14.6 ± 2.7 g/kg/day), followed by GSD patients (10.9 ± 1.4 g/kg/day), whereas carbohydrate intake was similar between controls and PKU patients. Fat intake did not differ significantly among the groups (*p* = 0.140). Dietary adherence rates were comparable among the metabolic disease groups, with no statistically significant difference (*p* = 0.385). Likewise, no statistically significant differences were observed among the metabolic disease groups in daily calcium intake (*p* = 0.797) or daily vitamin D intake (*p* = 0.066), although OA patients had numerically lower vitamin D intake. The median vitamin D intake was 0 IU/day because a substantial proportion of participants had no recorded vitamin D supplementation during the 3-day dietary assessment period.

Biochemical analyses demonstrated significant alterations in bone metabolism parameters in patients. Serum calcium levels were significantly lower in PKU (9.4 ± 0.6 mg/dL), OA (9.1 ± 0.6 mg/dL), and GSD patients (9.2 ± 0.7 mg/dL) compared with controls (9.8 ± 0.1 mg/dL) (*p* < 0.001). Similarly, serum phosphorus levels were significantly reduced in all metabolic disease groups compared with healthy controls (*p* < 0.001).

ALP and PTH levels were significantly higher than controls in all metabolic disease groups (*p* < 0.001 for both parameters). Mean ALP levels were 450.6 ± 112.3 U/L in PKU patients, 451.3 ± 99.7 U/L in OA patients, and 388.2 ± 109.4 U/L in GSD patients, compared with 284.0 ± 39.7 U/L in controls. Similarly, mean PTH levels were higher in PKU (56.6 ± 23.1 pg/mL), OA (65.0 ± 16.2 pg/mL), and GSD patients (55.0 ± 19.2 pg/mL) than in controls (39.9 ± 4.1 pg/mL).

Serum 25-hydroxyvitamin D concentrations were significantly lower in all inherited metabolic disease groups than in controls (*p* < 0.001). Mean vitamin D levels were 32.2 ± 1.8 ng/mL in controls, whereas values were 16.7 ± 9.3 ng/mL in PKU patients, 17.6 ± 15.3 ng/mL in OA patients, and 18.4 ± 14.4 ng/mL in GSD patients.

Among bone turnover markers, significant differences were observed in P1NP and osteocalcin levels. Mean P1NP concentrations significantly differed among the groups (*p* = 0.006), with the highest levels detected in PKU patients (677.1 ± 338.7 ng/mL). Lower P1NP concentrations were observed in OA (545.2 ± 320.4 ng/mL) and GSD patients (401.6 ± 190.9 ng/mL), while controls had a mean value of 470.8 ± 211.6 ng/mL.

Osteocalcin levels also significantly differed among the groups (*p* = 0.028). PKU patients had the highest osteocalcin concentrations (92.6 ± 39.4 ng/mL), whereas the lowest levels were observed in the GSD group (65.6 ± 24.4 ng/mL). Mean osteocalcin levels were 69.8 ± 27.4 ng/mL in controls and 78.7 ± 38.2 ng/mL in OA patients. In contrast, serum CTX levels did not significantly differ among the study groups (*p* = 0.183). Mean CTX levels were 1086 ± 499 pg/mL in controls, 1346 ± 569 pg/mL in PKU patients, 1104 ± 475 pg/mL in OA patients, and 1095 ± 351 pg/mL in GSD patients (Table 3).

DXA Z-scores differed significantly among the study groups. Mean DXA Z-scores were 0.2 ± 1.4 in controls, −0.1 ± 1.0 in PKU patients, −0.7 ± 1.2 in OA patients, and −1.1 ± 1.2 in GSD patients, with the lowest values observed in the GSD group.

## 4. Discussion

IMDs are increasingly recognized as chronic multisystemic conditions in which long-term complications extend beyond acute metabolic decompensation and neurological involvement. Advances in newborn screening, dietary treatment, and supportive care have significantly improved survival and quality of life in affected children; however, prolonged survival has also revealed chronic complications involving growth, nutrition, endocrine balance, and skeletal health [1,3,7]. Bone disease in IMDs is considered multifactorial and may result from restrictive therapeutic diets, altered nutrient composition, chronic metabolic instability, recurrent catabolic episodes, metabolic acidosis, endocrine dysregulation, and reduced physical activity [11,14]. In the present study, we observed significant differences in bone-related biochemical parameters and DXA Z-scores among children with PKU, OA, and GSD receiving long-term dietary treatment. Although PKU, OA, and hepatic GSD differ substantially in their underlying pathophysiology, disease-specific complications, and nutritional management, they share long-term dietary treatment as a cornerstone of therapy, which may be associated with shared nutritional challenges relevant to bone health. Therefore, our findings should be interpreted within the context of these shared nutritional challenges rather than as evidence of a common disease mechanism.

The main findings of our study included significantly lower vitamin D, calcium, and phosphorus levels, higher ALP and PTH levels than controls, and alterations of bone formation markers. Furthermore, among bone turnover markers, P1NP and osteocalcin were significantly altered, whereas CTX levels remained comparable among the groups, indicating different between-group patterns for the evaluated formation and resorption markers.

One of the most prominent findings of our study was the significantly lower serum 25-hydroxyvitamin D levels observed in all IMD groups. Vitamin D deficiency has consistently been reported in children and adults with inherited metabolic diseases and represents one of the major contributors to impaired skeletal mineralization [11,22]. The pathogenesis of vitamin D deficiency in IMDs is complex and likely multifactorial. Restrictive dietary regimens may reduce intake of natural vitamin D-containing foods, while recurrent illness, reduced outdoor activity, altered intestinal absorption, and long-term dependence on synthetic medical formulas may further compromise micronutrient balance [8,9]. In our cohort, reduced vitamin D levels were accompanied by elevated PTH levels, suggesting secondary hyperparathyroid responses aimed at maintaining calcium homeostasis. Persistent elevation of PTH may contribute to increased skeletal remodeling and progressive bone mineral loss over time [24,25,26]. The absence of significant differences in reported vitamin D intake among patient groups suggests that factors beyond dietary intake, including disease-related metabolic alterations, limited sun exposure, or impaired vitamin D metabolism, may contribute to the observed deficiency.

We also observed significantly lower serum calcium and phosphorus levels in all metabolic disease groups compared with controls. Although values were mostly within low-normal ranges, these findings support the presence of chronic disturbances in mineral homeostasis. Calcium and phosphorus are essential for bone mineralization, and even mild chronic reductions may negatively influence peak bone mass acquisition during childhood and adolescence [27,28,29]. Since childhood represents the most critical period for skeletal development, long-term disturbances in mineral balance may predispose IMD patients to reduced bone strength and future fracture risk.

ALP levels were significantly higher in all disease groups than in controls, consistent with differences in bone remodeling. ALP is an important marker of osteoblastic activity and mineralization and is commonly elevated in states of increased skeletal turnover [30]. In our study, higher ALP and PTH levels than in controls were observed together with lower vitamin D concentrations, which may reflect differences in bone remodeling and mineral homeostasis. Similar findings have been reported in previous studies evaluating skeletal health in PKU and GSD populations [8,17,22].

Among the evaluated bone turnover markers, the most remarkable findings involved P1NP and osteocalcin. P1NP is considered one of the most sensitive and reliable markers of bone formation, reflecting type I collagen synthesis during osteoblastic activity [31,32]. Osteocalcin is secreted by mature osteoblasts and is associated with overall bone turnover and bone matrix formation [33,34]. In our study, P1NP levels were higher in PKU patients than in controls and GSD patients, while osteocalcin levels were higher in PKU patients than in GSD patients.

Bone disease in PKU has been extensively investigated over the past decades. Multiple systematic reviews and meta-analyses have demonstrated lower BMD values in PKU patients compared with healthy populations [1,6,14,16]. However, the exact pathophysiological mechanisms remain incompletely understood. Several contributing factors have been proposed, including chronic dietary protein restriction, insufficient intake or bioavailability of calcium and vitamin D, altered amino acid metabolism, reduced physical activity, and chronic low grade metabolic acidosis related to amino acid-based medical formulas [10,11,23,25]. Rovelli et al. suggested that high potential renal acid load (PRAL) associated with amino acid mixtures may promote calcium mobilization from bone in PKU patients [11]. Furthermore, experimental studies have demonstrated alterations in osteoclastogenesis and RANKL/OPG signaling pathways in PKU, potentially contributing to remodeling imbalance [4,12]. However, acid-base status was not evaluated in the present study; therefore, this mechanism could not be directly assessed. The observed differences in P1NP and osteocalcin levels, together with lower DXA Z-scores, may reflect differences in bone remodeling in PKU patients.

Despite the between-group differences observed in bone formation markers, CTX levels did not differ significantly among the study groups. CTX reflects osteoclast-mediated bone resorption and is one of the most widely used markers of bone breakdown [7,31]. The absence of a significant between-group difference in CTX does not support a clear difference in bone resorption as reflected by this marker. Instead, our findings may reflect remodeling imbalance characterized by increased or dysregulated bone formation activity. Another possible explanation is the high physiological variability of CTX levels during childhood due to growth velocity and pubertal status [6,35]. Since bone turnover markers naturally fluctuate during growth and puberty, interpretation of CTX values in pediatric populations should be approached cautiously. Furthermore, the substantial age-dependent physiological variability of CTX during childhood may have reduced its ability to discriminate differences between disease groups.

Organic acidemias constitute another high-risk group for metabolic bone disease. Since the OA cohort predominantly consisted of patients with methylmalonic acidemia, the present findings mainly reflect skeletal involvement associated with MMA. In OA patients, chronic protein restriction, recurrent metabolic decompensations, mitochondrial dysfunction, metabolic acidosis, renal impairment, and nutritional deficiencies may all contribute to impaired skeletal health [16,17,18,27,28]. Previous reports have documented osteopenia, osteoporosis, vertebral compression fractures, and pathological fractures in children and young adults with propionic acidemia and methylmalonic acidemia [16,17]. In our cohort, OA patients had lower anthropometric measurements and lower calcium, phosphorus, and vitamin D levels than controls, together with higher ALP and PTH levels. These findings indicate between-group differences in anthropometric and bone-related biochemical parameters in the OA group. However, the cross-sectional design does not allow conclusions regarding the mechanisms underlying these differences.

GSD patients exhibited the lowest DXA Z-scores among all groups in our study. Because the majority of the GSD cohort consisted of patients with GSD type Ia, the observed findings should primarily be interpreted within the context of hepatic GSD type Ia. Skeletal involvement in hepatic glycogen storage diseases has previously been associated with chronic lactic acidosis, hyperlipidemia, impaired growth, delayed puberty, hormonal imbalance, and reduced muscle mass [19,20,21,36]. Particularly in GSD type Ia, chronic metabolic acidosis may impair mineralization and calcium balance. In GSD type III, muscle involvement and reduced mechanical loading are believed to contribute significantly to reduced bone mass through disruption of the muscle–bone relationship and impaired mechanostat function [21,22]. Reduced IGF-1 and insulin activity may further impair osteoblast maturation and bone formation [19,21]. In our cohort, GSD patients had lower weight and BMI than controls and PKU patients, lower height than PKU patients, and lower DXA Z-scores than controls. The higher protein intake observed in OA and GSD patients reflects disease-specific nutritional management aimed at preventing catabolism and maintaining metabolic stability. Nevertheless, differences in protein quantity and source may be associated with variations in bone metabolism. Whether these differences in protein intake are associated with long-term skeletal outcomes requires further investigation.

Anthropometric impairment itself may also contribute to reduced bone density in IMDs. OA patients had significantly lower height, weight, and BMI values than controls and PKU patients. GSD patients had significantly lower weight and BMI values than controls and PKU patients, whereas their height was significantly lower only than that of PKU patients. Since DXA measurements in children are influenced by body size and bone dimensions, short stature may lead to underestimation of areal BMD [37,38,39]. Differences in body size among the study groups may have influenced pediatric DXA measurements. Therefore, the DXA findings should be interpreted cautiously together with the observed biochemical differences.

However, several limitations should be acknowledged. First, this was a cross-sectional, single-center study with a relatively small sample size, particularly within individual disease subgroups. In addition, the study included biologically heterogeneous disorders with distinct underlying pathophysiological mechanisms, disease-specific complications, and nutritional management strategies that may influence bone metabolism differently. Therefore, our findings should be interpreted in the context of the shared nutritional challenges associated with long-term dietary treatment rather than as evidence of common mechanisms of skeletal involvement across these disorders. Larger multi-center studies with disease-specific analyses are warranted to better characterize skeletal involvement in individual inherited metabolic disorders. Second, bone turnover markers vary according to age, growth velocity, and pubertal status during childhood. Although the study groups were comparable with respect to age, Tanner staging data were unavailable for all participants. Third, DXA measurements in children may be influenced by short stature and body composition, particularly in disorders associated with impaired growth.

## 5. Conclusions

Children with inherited metabolic disorders receiving long-term dietary treatment showed lower vitamin D, calcium, and phosphorus levels and higher ALP and PTH levels than controls, together with differences in bone formation markers and lower DXA Z-scores. Bone formation markers showed more pronounced between-group differences than the bone resorption marker CTX. These findings emphasize the importance of regular biochemical and radiological monitoring of bone health in pediatric IMD populations and highlight the need for appropriate nutritional and metabolic management.

## Figures and Tables

**Table 1:** Demographic, anthropometric, dietary, biochemical, and bone metabolism characteristics of the study population.

		Min–Max			Median	Mean ± SD/n-%		
Age (year)		3.0	–	16.0	7.0	7.0	±	2.7
Age at the diagnosis (month)		1.0	–	48.0	3.0	6.7	±	9.1
Gender	Female					42		44.2%
	Male					53		55.8%
Height (cm)		90.0	–	170.0	116.0	119.0	±	17.4
Weight (kg)		12.0	–	58.0	20.0	22.8	±	8.5
BMI (kg/m^2^)		13.6	–	20.0	15.3	15.5	±	1.2
Daily energy (kcal/kg)		55.0	–	126.0	80.0	83.1	±	15.6
Protein (gr/kg/day)		1.00	–	2.50	1.40	1.57	±	0.53
Carbohydrate (g/kg/day)		7.0	–	19.0	10.0	10.8	±	2.7
Fat (g/kg/day)		2.0	–	3.5	2.8	2.8	±	0.3
Daily calcium intake (mg/day)		700.0	–	1300.0	1000.0	987.7	±	157.6
Daily vitamin D intake (IU)		0.0	–	1200.0	0.0	253.2	±	324.2
Serum Ca (mg/dL)		8.10	–	10.40	9.70	9.43	±	0.58
Serum phosphorus (mg/dL)		3.60	–	5.70	4.60	4.56	±	0.50
ALP (U/L)		210.0	–	670.0	364.0	385.0	±	116.1
PTH (pg/mL)		18.0	–	96.0	46.0	52.7	±	19.0
25 (OH) D (ng/mL)		4.0	–	56.0	22.0	22.2	±	12.7
CTX (pg/mL)		143.0	–	2225.0	1105.0	1160.2	±	492.2
P1NP (ng/mL)		95.3	–	1200.0	510.0	526.2	±	285.7
Osteocalcin (ng/mL)		18.0	–	167.0	77.1	76.8	±	33.9
DXA- Z Score		−2.70	–	4.00	0.10	0.38	±	1.73
Group	Control					30		31.6%
	PKU					25		26.3%
	OA					20		21.1%
	GSD					20		21.1%

Abbreviations: ALP, alkaline phosphatase; BMI, body mass index; Ca, calcium; CTX, C-terminal telopeptide of type I collagen; DXA, dual-energy X-ray absorptiometry; GSD, glycogen storage disease; OA, organic acidemia; P1NP, procollagen type I N-terminal propeptide; PKU, phenylketonuria; PTH, parathyroid hormone; SD, standard deviation; 25(OH)D, 25-hydroxyvitamin D.

**Table 2:** Comparison of demographic and anthropometric characteristics among the study groups.

			^1^ Control Group (n:30)			^2^ PKU (n:25)			^3^ OA (n:20)			^4^ GSD (n:20)			*p*	
Age	Average ± SD		7.22	±	2.39	7.92	±	3.32	6.10	±	2.34	6.53	±	2.13	0.104	^A^
	Median		7.00			7.00			6.00			6.75				
Age at the diagnosis (month)	Average ± SD					1.20	±	0.50	4.10	±	2.88	16.15	±	11.33	<0.001	^K^
	Median					1.00 ^3,4^			3.50 ^4^			12.00				
Gender	Female	n-%	15		50.0%	9		36.0%	10		50.0%	8		40.0%	0.681	**χ** ^2^
	Male	n-%	15		50.0%	16		64.0%	10		50.0%	12		60.0%		
Height	Average ± SD		122.7	±	14.9	126.7	±	19.3	109.6	±	15.3	113.0	±	14.7	0.002	^A^
	Median		123.5			127.0			107.0 ^1,2^			112.0 ^2^				
Weight	Average ± SD		24.2	±	6.5	27.2	±	11.1	18.4	±	6.0	19.5	±	6.4	<0.001	^K^
	Median		23.0			25.0			17.0 ^1,2^			17.5 ^1,2^				
BMI	Average ± SD		15.7	±	0.7	16.0	±	1.6	15.0	±	0.8	14.9	±	1.1	0.001	^K^
	Median		15.6			16.0			14.8 ^1,2^			14.9 ^1,2^				

Abbreviations: BMI, body mass index; GSD, glycogen storage disease; OA, organic acidemia; PKU, phenylketonuria; SD, standard deviation. Data are presented as mean ± SD, median, or n (%), as appropriate. ^A^: one-way analysis of variance (ANOVA); ^K^: Kruskal–Wallis test followed by pairwise Mann–Whitney U tests with Holm adjustment for multiple comparisons.; **χ**^2^: chi-square test. Superscript numbers indicate statistically significant pairwise differences based on adjusted *p*-values (*p* < 0.05): ^1^ vs. control group, ^2^ vs. PKU group, ^3^ vs. OA group, and ^4^ vs. GSD group.

**Table 3:** Comparison of biochemical parameters, bone turnover markers, and bone mineral density among the study groups.

		^1^ Control Group (n:30)			^2^ PKU Group (n:25)			^3^ OA Group (n:20)			^4^ GSD Group (n:20)			*p*	
Serum Ca (mg/dL)	Average ± SD	9.8	±	0.1	9.4	±	0.6	9.1	±	0.6	9.2	±	0.7	<0.001	^K^
	Median	9.8			9.4 ^1^			9.0 ^1^			9.1 ^1^				
Serum Phosphorus (mg/dL)	Average ± SD	4.9	±	0.2	4.4	±	0.5	4.4	±	0.5	4.5	±	0.6	<0.001	^K^
	Median	4.9			4.2 ^1^			4.3 ^1^			4.3 ^1^				
ALP (U/L)	Average ± SD	284.0	±	39.7	450.6	±	112.3	451.3	±	99.7	388.2	±	109.4	<0.001	^A^
	Median	282.5 ^2,3,4^			468.0			461.5			368.0				
PTH (pg/mL)	Average ± SD	39.9	±	4.1	56.6	±	23.1	65.0	±	16.2	55.0	±	19.2	<0.001	^K^
	Median	40.0 ^2,3,4^			56.0			69.0			52.5				
25 (OH) D (ng/mL)	Average ± SD	32.2	±	1.8	16.7	±	9.3	17.6	±	15.3	18.4	±	14.4	<0.001	^K^
	Median	32.0			16.0 ^1^			12.0 ^1^			11.0 ^1^				
CTX (pg/mL)	Average ± SD	1086	±	499	1346	±	569	1104	±	475	1095	±	351	0.183	^A^
	Median	1062			1408			1116			1050				
P1NP (ng/mL)	Average ± SD	470.8	±	211.6	677.1	±	338.7	545.2	±	320.4	401.6	±	190.9	0.006	^A^
	Median	499.5 ^2^			646.0			542.5			362.0 ^2^				
Osteocalcin (ng/mL)	Average ± SD	69.8	±	27.4	92.6	±	39.4	78.7	±	38.2	65.6	±	24.4	0.028	^A^
	Median	71.9			96.3			81.7			62.6 ^2^				
DXA Z Score	Average ± SD	0.2	±	1.4	−0.1	±	1.0	−0.7	±	1.2	−1.1	±	1.2	<0.001	^A^
	Median	0.4			0.0 ^1^			−0.7 ^1^			−1.2 ^1^				

Abbreviations: ALP, alkaline phosphatase; Ca, calcium; CTX, C-terminal telopeptide of type I collagen; DXA, dual-energy X-ray absorptiometry; GSD, glycogen storage disease; OA, organic acidemia; P, phosphorus; P1NP, procollagen type I N-terminal propeptide; PKU, phenylketonuria; PTH, parathyroid hormone; SD, standard deviation; 25(OH)D, 25-hydroxyvitamin D. Data are presented as mean ± SD or median, as appropriate. ^A^: one-way analysis of variance (ANOVA); ^K^: Kruskal–Wallis test followed by pairwise Mann–Whitney U tests with Holm adjustment for multiple comparisons. Superscript numbers indicate statistically significant pairwise differences based on adjusted *p*-values (*p* < 0.05): ^1^ vs. control group, ^2^ vs. PKU group, ^3^ vs. OA group, and ^4^ vs. GSD group.

## Data Availability

The original contributions presented in the study are included in the article and its Supplementary Material. Further inquiries can be directed to the corresponding author (Sabire Gokalp).

## Supplementary Materials

The following supporting information can be downloaded at https://www.mdpi.com/article/10.3390/nu18172751/s1: Table S1: Comparison of dietary intake among the study groups.
