# Supplementary material for: Biochemical Evaluation of Bone Health in Children with Phenylketonuria, Organic Acidemias, and Glycogen Storage Diseases Under Dietary Management
Source: Nutrients. 2026 Aug 22;18(17):2751. doi: 10.3390/nu18172751 (PMC13567019; doi:10.3390/nu18172751)
Supplement: Supplementary file 1 [file nutrients-18-02751-s001.zip › nutrients-4452519-supplementary.pdf]

Supplementary Table S1. Comparison of dietary intake among the study groups.

		[1]Control group (n:30)	[2]PKU Group (n:25)	[3]OA Group (n:20)	[4]GSD Group (n:20)	p
Daily energy (kcal/kg/day)	Average.±SD	75.3 ± 7.6	72.8 ± 9.0	96.8 ± 16.0	93.9 ± 14.3	***0.000*** K
		78.0^{34}	70.0^{34}	100.0	100.0	
	Median		0.30	0.56		
Protein (g/kg/day)	Average.±SD	1.04 ± 0.05	1.38 ± 0.19	1.94 ± 0.38	2.25 ± 0.27	***0.000*** K
	Median	1.00^{234}	1.40^{34}	2.00^{4}	2.20	
Carbohydrate (g/kg/day)	Average.±SD	9.4 ± 1.3	9.2 ± 1.2	14.6 ± 2.7	10.9 ± 1.4	***0.000*** K
	Median	9.5^{34}	9.0^{34}	15.2	10.0^{3}	
Fat(g/kg/day)	Average.±SD	2.7 ± 0.2	2.9 ± 0.3	2.7 ± 0.4	2.7 ± 0.3	0.140 K
	Median	2.7	3.0	2.8	2.6	
Daily calcium intake (mg/day)	Average.±SD	980 ± 145	1020 ± 135	960 ± 182	975 ± 159	0.797 K
	Median	1000	1000	1000	1000	
Daily vitamin D intake (IU/day)	Average.±SD	340.0 ± 311.4	291.7 ± 382.1	100.0 ± 210.3	230.0 ± 326.2	0.066 K

Abbreviations: A, one-way ANOVA; K, Kruskal–Wallis test with Mann–Whitney U test for pairwise comparisons; χ^2, chi-square test. Values are presented as mean ± SD or median, unless otherwise indicated. Superscript numbers indicate statistically significant pairwise differences ($p < 0.05$): [1] vs. Control group, [2] vs. PKU group, [3] vs. OA group, and [4] vs. GSD group.
